# Thermal Degradation Behavior of Epoxy Resin Containing Modified Carbon Nanotubes

**DOI:** 10.3390/polym13193332

**Published:** 2021-09-29

**Authors:** Xiaohui Bao, Fangyi Wu, Jiangbo Wang

**Affiliations:** School of Materials and Chemical Engineering, Ningbo University of Technology, Ningbo 315211, China; bxh19883979039@163.com (X.B.); w18758809619@163.com (F.W.)

**Keywords:** epoxy resin, carbon nanotubes, silicone, thermal degradation, flame retardancy, activation energy

## Abstract

Via the surface-grafting of carbon nanotubes (CNTs) with a silicon-containing flame retardant (PMDA), a novel flame retardant CNTs-PMDA was synthesized. The flame retardancy was tested by cone calorimeter. Compared with pure epoxy resin, the total heat release (THR) and peak heat release rate (PHRR) of epoxy resin containing CNTs-PMDA were significantly reduced, by 44.6% and 24.6%, respectively. Furthermore, thermal degradation behavior of epoxy resin based composite was studied by the thermogravimetric analysis with differences in heating rates. The kinetic parameters of the thermal degradation for epoxy resin composites were evaluated by the Kissinger method and Flynn-Wall-Ozawa method. The results suggested that activation energy values of epoxy resin containing CNTs-PMDA in thermal degradation process were higher than those of pure epoxy resin in the final stage of the thermal degradation process, which was closely related to the final formation of char layer residues. Finally, the results from Dynamic mechanical thermal analysis (DMTA) and Scanning electron microscopy (SEM) measurements exhibited that the functionalization of CNTs with PMDA obviously improved the dispersion of CNTs in the epoxy resin matrix.

## 1. Introduction

Epoxy resins (EP) have been developed for nearly 70 years; their applications cover such varied fields as composite matrices, surface coatings, adhesives, and encapsulation of electronic components, as well as the aeronautical and astronautical industries. Features of epoxy resins such as their excellent mechanical properties, good chemical and moisture resistance, long-term service time, low cost and easy processing are often attractive [1,2,3,4]. Nevertheless, the inherent flammability of EP limits its application in many fields due to safety concerns. Therefore, flame retardants have to be used in order to fulfill the requirements of flammability tests [5,6,7].

In general, carbon nanotubes (CNTs) have been of interest to researchers because they frequently exhibit superior flame retardancy, mechanical and electronic properties. Advanced improvements when compared with conventional flame retardant containing halogen, they are free of toxic smoke or corrosive fumes during combustion [8,9,10]. By adding 2 wt% carbon nanotube flame retardant to polymer, flame retardancy can be significantly improved without damaging other properties of the polymer. Moreover, carbon nanotubes can also be prepared by extracting lignin from bamboo charcoal, rice straw, coconut fibre and corn straw as carbon sources. Therefore, it has excellent environmentally friendly properties and recyclability [11,12,13]. However, the agglomeration of CNTs owing to strong van der Waals forces and π–π interactions between carbon nanotubes make it quite impossible to realize the ideal functions of CNTs in polymer nanocomposites [14,15]. To enhance interfacial adhesion between nanotubes and the polymer matrix, chemical functionalization of carbon nanotubes is one of the applicable methods. It can be concluded that the polymers grafted onto CNTs could desirably improve their dispersion but also simultaneously destroy the flame retardancy of the materials due to the polymer’s inherent flammability [16,17]. Nowadays, solving these problems usually requires the functionalization of CNTs using some reactive flame retardants such as fullerene, intumescent flame retardant, etc. [17,18,19,20]. On the other hand, thermal degradation kinetic methods are widely used to analyze the combustion processes of polymers, including epoxy resin, polycarbonate, polyvinyl chloride, etc. [21,22,23]. Understanding the role of flame retardants in the combustion process of polymers is helpful to reveal their flame retardant mechanism.

In this article, a novel silicon-containing flame retardant (PMDA), which was composed of 35 mol% methylsiloxane, 60 mol% phenylsiloxane and 5 mol% aminosiloxane units, was prepared by hydrolysis and polycondensation. Then, the PMDA was grafted onto the surface of the CNTs, and the epoxy resin/CNTs-PMDA nanocomposites were subsequently prepared. The flame retardancy and thermal degradation behavior of the flame-retardant epoxy resins were determined by the cone calorimeter and thermogravimetric analysis (TGA), respectively, for the purpose of gaining insight into the possible flame retardant mechanism.

## 2. Materials and Methods

### 2.1. Materials

Methyltrimethoxysilane (MTMS, 98%), tetramethylammonium hydroxide (TMAOH, 97%), pyridine (99.5%), tetrahydrofuran (THF, 99%), concentrated sulphuric acid (H_2_SO_4_, 98%), nitric acid (HNO_3_, 65–70%), (3-aminopropyl)trimethoxysilane (APS, 97%) and N,N-dimethylformamide (DMF, 99%) were all obtained from Alfa Aesar Chemical Reagent Co. Ltd. (Tewksbury, MA, USA). Dimethyldimethoxysilane (DMDS, 95%), phenyltrimethoxysilane (PTMS, 97%) were both reagent grade and provided by Gelest Chemical Reagent Co. Ltd. (Morrisville, PA, USA). Ethyl alcohol (EtOH, 95%) was supplied by Sigma-Aldrich Reagent Co. Ltd. (St. Louis, MO, USA). Thionyl chloride (SOCl_2_, 98%) and chloroform (CHCl_3_, ≥99%) were purchased from Fisher Scientific Chemical Co. (Waltham, MA, USA). EPON 826 containing an epoxy equivalent weight of 178–186g was offered by Hexion Specialty Chemicals Inc. (Columbus, OH, USA) and used as received. The hardener with an amine equivalent weight of 60 g, Jeffamine D230, was purchased from Huntsman Corp. (Woodlands, TX, USA) and also used as received. CNTs (length at 10–30 μm, outer diameter at 10–20 nm, inner diameter at 5–10 nm,) synthesized by chemical vapor deposition were supplied by Chengdu Organic Chemistry Co. Ltd., Chinese Academy of Science (Sichuan, China). It was composed of 99.4% carbon nanotubes and 0.6% catalyst Ni, and contained no conventional carbon products such as graphite and carbon black.

### 2.2. Synthesis of Polysilicone (PMDA)

**Scheme 1:**
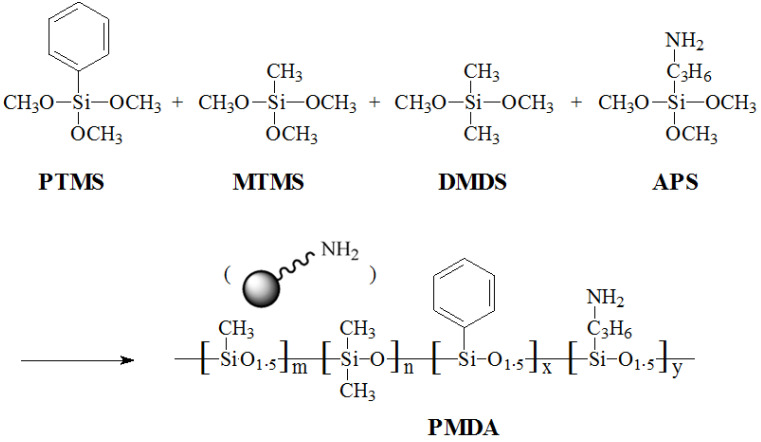
Synthesis route of PMDA.

The polysilicone PMDA was synthesized by the hydrolysis and polycondensation method as shown in Scheme 1. The raw material consisted of 60 mol% phenylsiloxane, 35 mol% methylsiloxane and 5 mol% aminosiloxane as a unit. The ratio of organic groups to silicon atoms (R/Si) was 1.2, which was used to characterize the extent of branches to a polysiloxane structure; the molecular structure of polysilicone is illustrated in Scheme 1. Distilled water (25 mL), EtOH (75 mL) and TMAOH (1 mL) were mixed in a 250 mL flask under stirring, followed by adding the mixture of PTMS, MTMS, DMDS and APS at certainly molar ratios (0.69:0.06:0.20:0.05) and maintaining 10% weight percentage solution. The stirring was maintained for 8 h, and the resulting solution was stored at room temperature overnight. Through decantation of most clear supernatant, precipitated condensate was collected and then washed by vacuum filtration with distilled H_2_O/EtOH (1/3 by volume), then washed again in pure EtOH. Finally, the acquired rinsed powder (PMDA) was thoroughly dried under vacuum for 20 h at room temperature [24].

### 2.3. Functionalization of CNTs

The synthesis steps of CNTs-COCl were as follows: The mixture of HNO_3_ (30 mL), H_2_SO_4_ (90 mL) and CNTs, was sonicated at 50 °C for 2 h and, after termination of the reactioncould be cooled to room temperature. The mixture was diluted with deionized water, then underwent vacuum filtering using a nylon film (0.22 μm, Sangon Biotech Co. Ltd., Shanghai, China). The obtained solid was CNTs-COOH, whose polar carboxyl groups were successfully introduced into the convex surface of CNTs, then washed with a large amount of deionized water until the aqueous layer reached neutral. The solid was vacuum dried at 80 °C for 12 h. In the next step, the reaction mixture of CNTs-COOH (200 mg), SOCl_2_ (20 mL) and DMF (1 mL) was sonicated at 50 °C for 1 h, after which the reflux was maintained at 70 °C for 24 h. Finally, the temperature was raised to 120 °C to remove residual SOCl_2_ via reduced pressure distillation, and CNTs-COCl correspondingly obtained.

**Scheme 2:**
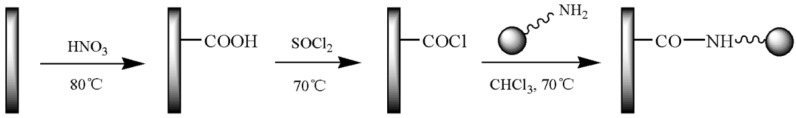
Illustration for the functionalization of the CNTs with PMDA.

In the next step, the synthesis of CNTs-PMDA was as follows (Scheme 2): the suspension of CNTs-COCl (100 mg) and DMF (50 mL) was introduced by PMDA (400 mg) and pyridine (1 mL, as cat.), under the protection of nitrogen. The mixture was reacted at 70 °C for 24 h. When cooling to room temperature, the dark solution was filtered and washed to remove unreacted PMDA. The target product, CNTs-PMDA, was obtained after vacuum drying at 80 °C for 24 h [24].

### 2.4. Preparation of Epoxy Composites

Briefly, the EP/CNTs-PMDA composite was prepared as follows: the above semi-product, CNTs-PMDA (2 g), was dispersed in acetone (100 mL) and sonicated for 1 h until forming a uniform black suspension. EPON 826 (73.5 g) was added into the mixture and then mechanically dispersed through stirring for 30 min. The mixture was heated in a vacuum oven at 50 °C for 10 h to remove the solvent thoroughly. Subsequently, D230 (24.5 g) was added into the mixture while stirring was maintained for 30 min [25]. The composite was put into vacuum again tobe degassed for 10 min, in order to remove any trapped air. Finally, the sample was cured at 80 °C and post cured at 135 °C for 2 h, respectively. For comparison, pure EP was also prepared underthe same processing conditions [26].

### 2.5. Characterization and Measurement

The Fourier transform infrared spectroscopy (FTIR) spectra was tested using a Digilab Scimitar FTS-2000 IR spectrometer (Digilab Inc., Hopkinton, MA, USA). The surface morphology of carbon nanotubes was observed by JEOL JEM-2100F transmission electron microscopy (TEM, JEOL Ltd., Akishima-shi, Tokyo, Japan). Cone calorimeter measurement was performed on an FTT cone calorimeter (Fire Testing Technology Ltd., East Grinstead, West Sussex, UK) according to ASTM E1354. The heat flux was 50 kW/m^2^. The dimensions of each specimen were 100 × 100 × 3 mm^3^. Thermogravimetric analysis (TGA) was carried on a TA instrument Q5000 thermogravimetric analyzer (TA Instrument Corp., New Castle, DE, USA). The sample (about 10 mg) was heated from 50 °C to 600 °C in a nitrogen atmosphere. Dynamic mechanical thermal analysis (DMTA) was determined using a Rheometric Scientific SR-5000 dynamic mechanical analyzer (Rheometric Scientific Inc., West Yorkshire, UK), and the data were collected from 40 °C to 140 °C at a scanning rate of 5 °C/min. The samples were coated with a conductive gold layer and examined by scanning electron microscopy (SEM) using an FEI Quanta 200 environmental scanning electron microscope (FEI Co., Hillsboro, OR, USA).

### 2.6. Thermal Degradation Theory

The reaction rate of thermal transformation of a solid state chemical reaction based on the assumption is:

(1) $$r=\frac{da}{dt}=kf(a)$$

where *f*(*a*) is the assumed reaction model, *a* is the degree of conversion, *k* the temperature dependent rate constant, *t* the time and *r* the rate of degradation. *k* is normally presumed to conform to the Arrhenius equation:

(2) k=Aexp(−E/RT)

where *E* is the activation energy of the kinetic process, *A* the pre-exponential factor, *T* the temperature and *R* the universal gas constant.

The rate of degradation is dependent on the temperature and the weight change of the sample, and can be expressed as [26]:

(3) $$\frac{da}{dt}=Af(a)\exp(-E/RT)$$

Equation (3) is derived in its integral form, which particularly for isothermal conditions becomes

(4) lnt=E/RT−ln[A/g(x)]

Another condition for non-isothermal degradation, Equation (3) becomes

(5) $$\frac{da}{dT}=(A/\beta )f(a)\exp(-E/RT)$$

where *β* is the heating rate ($\beta =\frac{dT}{dt}$) and *g(x)* is the integrated form of mechanism ($g(x)=\int_{0}^{a}\frac{da}{f(a)}$).

#### 2.6.1. Kissinger Method

The Kissinger expression [27] is as follows:

(6) $$\ln(\frac{\beta }{T_{\max}^{2}})=\ln(\frac{AR}{E})-\frac{E}{RT_{\max}}$$

where *T*_max_ is the temperature of the peak rate.

From the above Equation (6), the peak rate temperatures determined at certain different heating rates allow the activation energy to be calculated by the Kissinger method. Plotting the natural logarithm of $\ln(\beta /T_{\max}^{2})$ against the reciprocal of the absolute temperature (1/*T*_max_), the slope of the resulting line is given by −*E/R*, which allows the value of *E* to be obtained [28].

#### 2.6.2. Flynn-Wall-Ozawa Method

The equation of the Flynn-Wall-Ozawa method [29,30] can be expressed as follows:

(7) $$\mathrm{lg}(\beta )=\mathrm{lg}AE/g(a)R-2.315-0.457\frac{E}{RT}$$

The above equation shows that lg(β) is linearly proportional to 1/*T*. The activation energy for any particular degree of degradation can then be determined by a calculation of the slope from the lg(β) − 1/*T* plots [28].

## 3. Results

### 3.1. Structural Characterization

**Figure 1:**
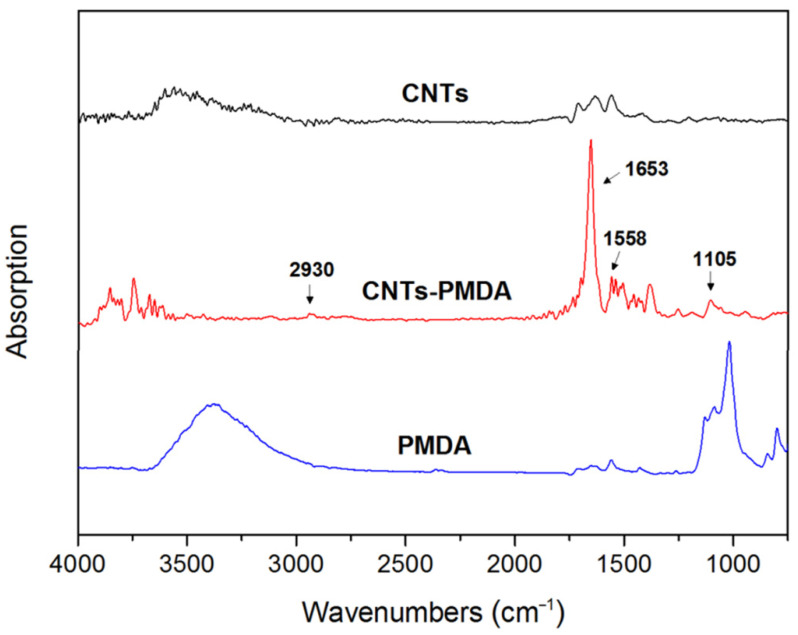
FTIR spectra of PMDA, CNTs and CNTs-PMDA.

Figure 1 exhibits and compares the FTIR spectra of PMDA, CNTs and CNTs-PMDA. As shown in the pristine CNT spectra at the top, the bands at around 1650 and 3500 cm^−1^ were attributed to the presence of carbonyl (quinone) and hydroxyl groups, respectively, which means that the CNTs contained some impurities [31,32]. For the middle spectrum, the reaction of CNTs with the PMDA led to the formation of an amide group, which exhibited close wavenumber bands with very close carboxylic groups (the strongpeak at 1653 cm^−1^ was attributed to the carbonyl in -COOH and -CONH- structures). The band at around 1558 cm^−1^ characteristic of a monosubstituted amide is evidence of the formation of an amide bond. Moreover, compared with the CNTs spectra other bands at 1105 cm^−1^ (Si-O-Si) and 2930 cm^−1^ (-CH_2_-) in CNTs-PMDA appeared, demonstrating that the PMDA had been grafted to the surface of the functionalized CNTs [24].

**Figure 2:**
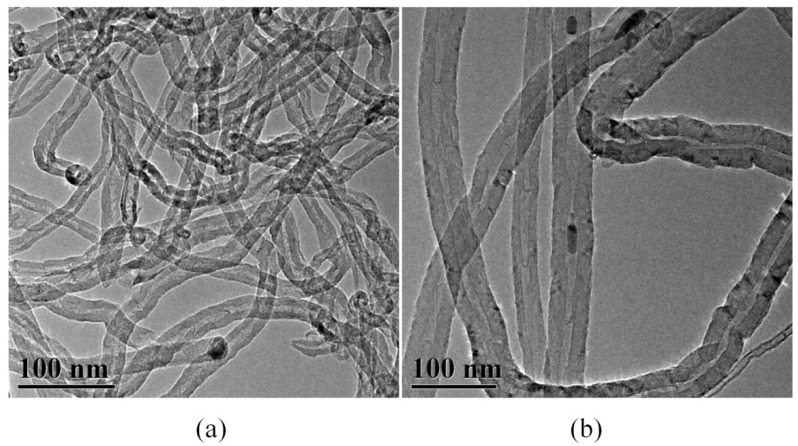
TEM images of CNTs (**a**) and CNTs-PMDA (**b**).

The TEM measurement of CNTs and CNTs-PMDA can provide direct morphological evidence for the grafting of PMDA onto the surface of the CNTs (Figure 2). As can be seen from Figure 2a, the carbon tube diameter of pristine CNTs was between 15~30 nm, and the surface was smooth and clean without any adhesion. In contrast, the surface of CNTs-PMDA in Figure 2 was rough, and its tube diameter was between 40~50 nm, which was significantly larger than that of pristine CNTs. Therefore, it can be proven from the appearance of CNTs and CNTs-PMDA that PMDA was indeed grafted onto the surface of the carbon tubes.

### 3.2. Flame Retardancy

**Figure 3:**
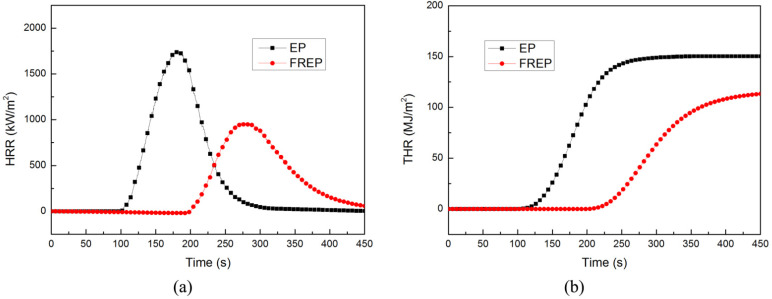
HRR (**a**) and THR (**b**) curves for EP composites.

Cone calorimeter measurement can provide a lot of information on the combustion properties of the studied materials. Figure 3 illustrated the heat release rate (HRR) and total heat release (THR) as functions of time for epoxy resin (EP) and EP/CNTs-PMDA (FREP). After ignition, pure EP exhibited a single peak of basic pairs in the HRR curve with a peak height of 1742 kW/m^2^. Presence of CNTs-PMDA in EP dramatically decreased the peak heat release rate (PHRR) value for EP by 44.6% and increased the time-to-PHRR (tPHRR). In addition, for the FREP the THR was also significantly reduced, by 24.6% compared with that of pure EP. This is attributed to the initial formation of the char layer, which inhibited the heat from pyrolyzing the underlying matrix.

### 3.3. Thermal Stability

**Figure 4:**
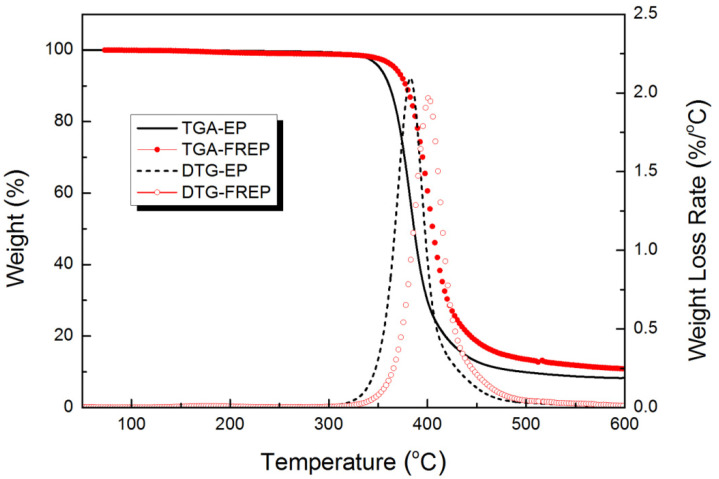
Thermal stability for EP composites.

The TGA and DTG curves of EP composites at 10 °C/min are depicted in Figure 4, and the relative data summarized in Table 1. Compared with pure EP, the temperatures of 5% mass loss (*T*_5wt%_) and the maximum mass loss rate (*T*_max_) of FREP were raised by 15.4 °C and 18.7 °C, respectively. The use of 5 wt% CNTs-PMDA caused the peak rate to decrease from 2.10 to 1.97 wt%/°C, which was helpfulfor improving the flame retardancy of EP with CNTs-PMDA. Furthermore, the addition of CNTs-PMDA led to an increase from 8.27 to 10.86 wt% in the residue char amounts. Therefore, the flame retardancy of EP was improved on the basis of CNTs-PMDA. This is further proven by the following activation energy data (Table 1).

### 3.4. Thermal Degradation Kinetics

**Figure 5:**
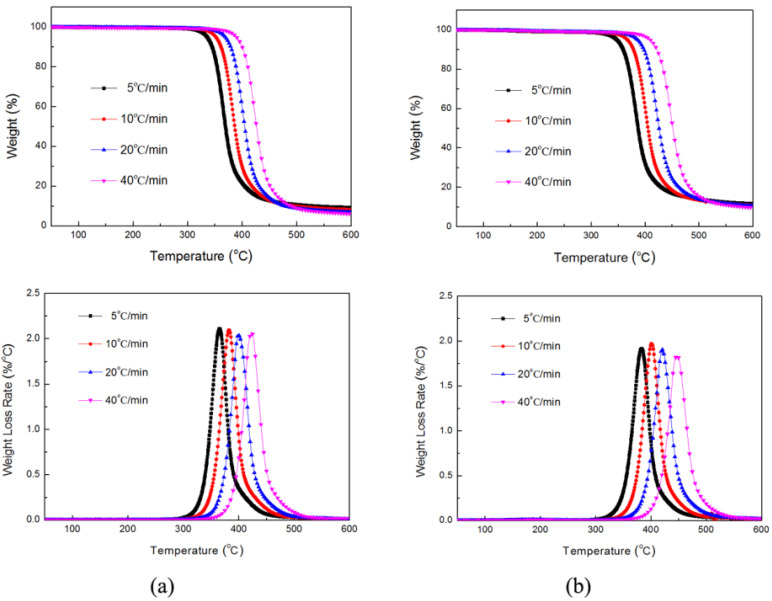
TGA and DTG curves of (**a**) EP and (**b**) FREP composites.

As a tool for unraveling the mechanisms of the physical and chemical processes that occur during polymer degradation, the application of non-isothermal TGA methods holds great promise. The TGA and DTG curves of EP composites at different heating rates (5, 10, 20, 40 °C/min) in nitrogen were depicted in Figure 5. It can be seen that with increasing heating rate, degradation started at higher temperatures. The reason for this transformation is that higher degradation temperatures at higher heating rates are due to the reduction in residence time, which is not sufficient for heat to permeate to the center of the reactants. Thus, the thermal decomposition process is delayed.

**Figure 6:**
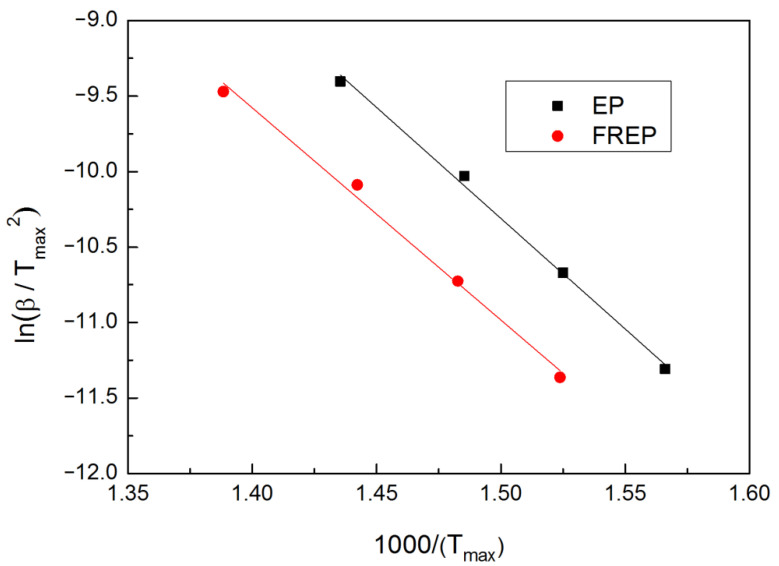
The curves of $\ln(\beta /T_{\max}^{2})$ vs. 1/*T*_max_ of EP and FREP.

As far as the results of the Kissinger method were concerned, the *E* and ln*A* values of degradation for the EP composites were determined from the slope and intercept of the $\ln(\beta /T_{\max}^{2})$ vs. 1/*T*_max_ linear equations (Figure 6), respectively, presented in Table 2.

It was be found that the *E* values of pure EP and FREP were 122.1 and 117.0 kJ/mol, respectively, which indicated that the addition of CNTs-PMDA led to a decrease in the value of the EP composites. The results confirmed that the flame retardant CNTs-PMDA induced the thermal degradation of polymer.

**Figure 7:**
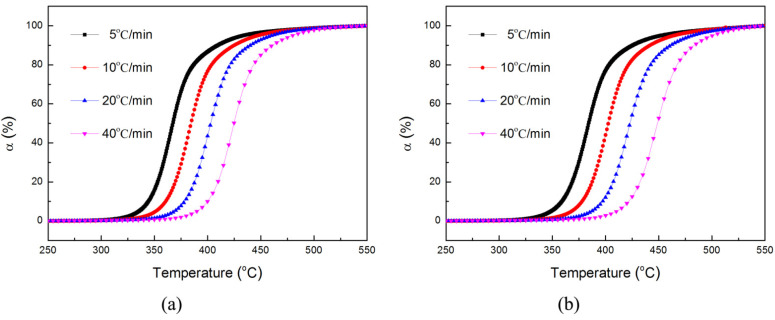
Conversion of (**a**) EP and (**b**) FREP in function of temperature.

For comparison purposes, the activation energies for the EP composites under thermal degradation were also calculated by the Flynn-Wall-Ozawa method. According to Equation (7), the temperature of specific conversion rates such as a = 2%, 5%, 10%, 20%, 30%, 40%, 50%, 60%, 70%, 80%, 90%, 95%, 98% can be achieved from the TGA curves at various heating rates; the results were then plotted in Figure 7. Figure 7 shows that with the increase of the heating rate at isoconversion rate, the temperature increased significantly.

**Figure 8:**
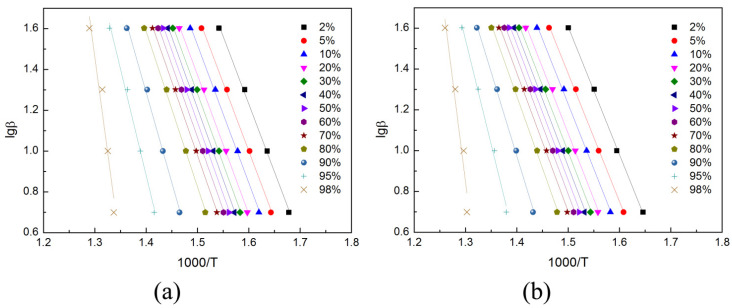
The curves of lg(β) vs. 1/T of EP (**a**) and FREP (**b**).

Figure 8 illustrates lg(β) vs. 1/*T*. The activation energy can be calculated from the slope of the plots of lg(β) vs. 1/*T* by Equation (7) with a conversion rate from 2% to 98%. According to the plots, there was a good linear relation between lg(β) and 1/*T*.

**Figure 9:**
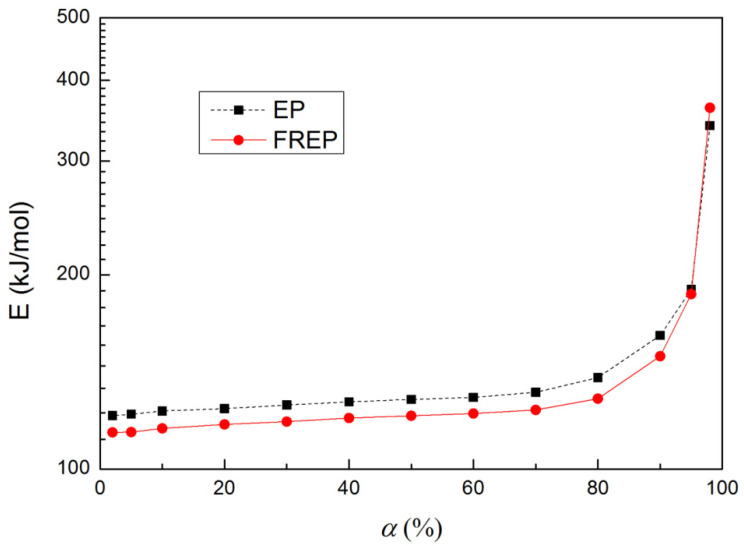
Activation energy curves of EP and FREP by Flynn-Wall-Ozawa method.

The values of *E* vs. *α* were exhibited in Figure 9. For pure EP, the changes of activation energies were minimal in the range of conversion rates from 0.02 to 0.80, so that kinetic parameters could be regarded as fixed. When *α* > 0.8, an obvious change of *E* occurred, which was related to the formation of the char layer and to the change in kinetic parameters. Compared with the *E* of pure EP, the variation of FREP had a similar law and the *E* of thermal degradation increased with the increase of the *α*. However, the *E* values of FREP were higher than that of pure EP in the final stage, which suggested that it was closely related to the formation of the final char residue layer.

This is consistent with the reports in the literature. The residue of pure EP had a lot of open holes with different sizes, which provided channels for the combustible volatiles from the inner matrix and for heat feedback from the flame [32]. After the functionalization of CNTs with flame retardants (such as silicone, phosphorus nitrogen compound), the residue char of FREP was very compact and dense, and few open holes were found. This can be explained by the wrapped CNTs forming a continuous network structured protective layer so that the entire structure could then effectively act as a barrier to limit the diffusion of flammable gases to the surface and slow down the combustion and degradation of the polymer [32,33,34].

### 3.5. Dispersion

**Figure 10:**
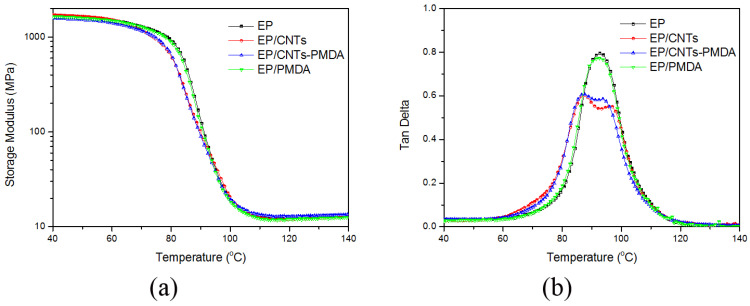
Dynamic storage modulus (**a**) and tan δ (**b**) of EP composites.

The mechanical properties of CNT-based composites are closely related to the dispersion of carbon tubes in the polymer matrix. Therefore, it is very important to study the dispersion of CNTs in the polymer matrix. DMTA and SEM measurements of composites can provide rich information in this regard. Figure 10 shows the DMTA curve of the EP composite. It can be seen that the tangent delta curves of EP/CNTs and EP/CNTs-PMDA exhibited a double-peak distribution, which indicates that there was an obvious microphase separation between the carbon tubes and polymer matrix. It was found that the distance between the two peaks of EP/CNTs-PMDA (6.0 °C) was less than that of EP/CNTs (9.4 °C), that is, the degree of phase separation was reduced. This showed that the grafting modification of PMDA on the surface of CNTs is conducive to improving the dispersion of carbon tubes in the polymer matrix.

**Figure 11:**
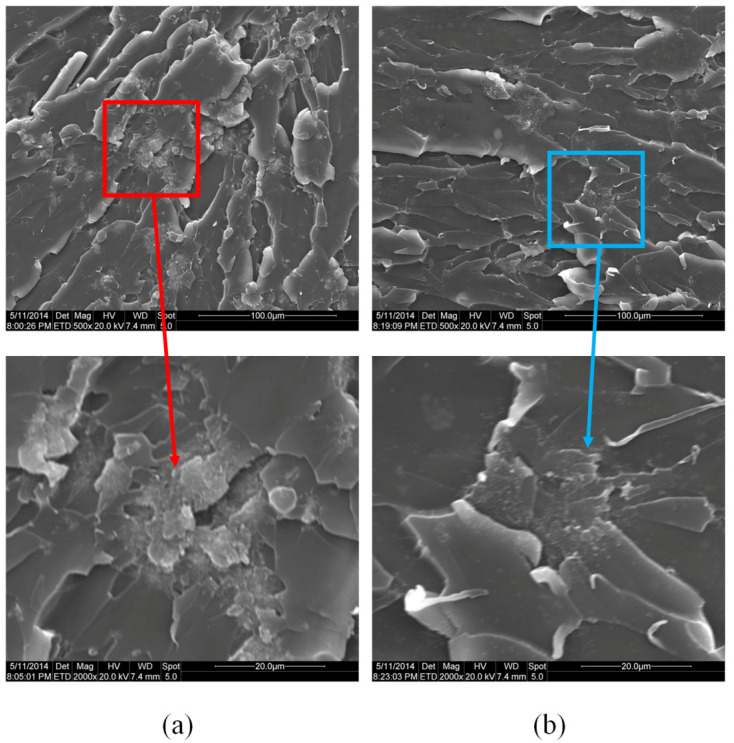
SEM images of (**a**) CNTs and (**b**) CNTs-PMDA in the EP matrix.

Figure 11 shows the SEM images of CNTs and CNTs-PMDA in the EP matrix; the carbon nanotube aggregate are indicated by the box. As shown in Figure 11a, it was evident that many large CNT aggregates were present in the EP nanocomposite, and the pristine CNT bundles or ropes are more obvious under higher magnification. In comparison, the SEM images in Figure 11b clearly displayed that CNTs-PMDA dispersed more homogeneously in the polymer matrix than the pristine CNTs in Figure 11a, although some nanotube agglomerates still appeared. This indicates that after being grafted with PMDA, the interfacial compatibility between CNTs and the EP matrix was remarkably enhanced. Subsequently, the dispersion of CNTs in the polymer matrix was improved, which was completely consistent with the study results of DMTA measurement.

## 4. Conclusions

In this paper, the flame retardant CNTs-PMDA was synthesized via the surface grafting of CNTs with PMDA. The results of cone calorimeter measurement indicated that the incorporation of CNTs-PMDA significantly decreased the peak heat release rate (PHRR) and total heat release (THR) of EP composite by 44.6% and 24.6%, respectively. Furthermore, kinetic studies using both Kissinger method and Flynn-Wall-Ozawa method revealed that a higher activation energy of FREP was obtained in the final stage compared with pure EP; this was related to the formation of the final residual char layer. Finally, the results from DMTA and SEM measurements exhibited that the functionalization of CNTs with PMDA obviously improved the dispersion of CNTs in the EP matrix.

## Figures and Tables

**Table 1:** TGA data of EP composites.

	Temperature (°C)		Peak Rate (wt%/°C)	Residual Char (wt%)
	*T*_5wt%_	*T*_max_		
EP	351.7	382.6	2.10	8.27
FREP	367.1	401.3	1.97	10.86

**Table 2:** Kinetic parameters for the degradation of EP and FREP by the Kissinger method.

	*T*_max_ (°C)				Fitting Equation	*R2* Values	*E* (kJ/mol)	ln*A* (1/min)
	5 °C/min	10 °C/min	20 °C/min	40 °C/min				
EP	365.4	382.6	400.1	423.5	Y = 11.72 − 14.69x	0.9948	122.1	14.4
FREP	383.1	401.3	420.2	447.1	Y = 10.11 − 14.01x	0.9906	117.0	12.8

## Data Availability

The data used to support the findings of this study are available from the corresponding author upon request.
